# Nature of the Electrochemical Properties of Sulphur Substituted LiMn_2_O_4_ Spinel Cathode Material Studied by Electrochemical Impedance Spectroscopy

**DOI:** 10.3390/ma9080696

**Published:** 2016-08-16

**Authors:** Monika Bakierska, Michał Świętosławski, Roman Dziembaj, Marcin Molenda

**Affiliations:** Faculty of Chemistry, Jagiellonian University, Ingardena 3, Krakow 30-060, Poland; monika.bakierska@gmail.com (M.B.); dziembaj@chemia.uj.edu.pl (R.D.)

**Keywords:** Li-ion battery, cathode material, LiMn_2_O_4_ spinel, sulphur substitution, electrochemical properties

## Abstract

In this work, nanostructured LiMn_2_O_4_ (LMO) and LiMn_2_O_3.99_S_0.01_ (LMOS1) spinel cathode materials were comprehensively investigated in terms of electrochemical properties. For this purpose, electrochemical impedance spectroscopy (EIS) measurements as a function of state of charge (SOC) were conducted on a representative charge and discharge cycle. The changes in the electrochemical performance of the stoichiometric and sulphur-substituted lithium manganese oxide spinels were examined, and suggested explanations for the observed dependencies were given. A strong influence of sulphur introduction into the spinel structure on the chemical stability and electrochemical characteristic was observed. It was demonstrated that the significant improvement in coulombic efficiency and capacity retention of lithium cell with LMOS1 active material arises from a more stable solid electrolyte interphase (SEI) layer. Based on EIS studies, the Li ion diffusion coefficients in the cathodes were estimated, and the influence of sulphur on Li^+^ diffusivity in the spinel structure was established. The obtained results support the assumption that sulphur substitution is an effective way to promote chemical stability and the electrochemical performance of LiMn_2_O_4_ cathode material.

## 1. Introduction

Lithium-ion batteries (LIBs) are the most prevalent power supplies for many portable electronic devices, owing to their low weight, high energy, and power density. Recently, LIBs have also attracted widespread interest in developing hybrid vehicles, plug-in hybrid vehicles, electric vehicles (xEV), and energy storage systems (ESS) [1,2,3,4,5].

Lithium-manganese oxide spinel (LiMn_2_O_4_, LMO) is regarded as a favourable cathode material for rechargeable Li-ion batteries. It has many advantages, such as the high abundance of manganese resources, low cost, environmental friendliness, high thermal stability, good safety features, and competitive theoretical capacity (about 148 mAh g^−1^) compared to layered lithium cobalt oxide (LiCoO_2_, LCO), lithium nickel oxide (LiNiO_2_, LNO), and their derivatives [6,7,8,9]. The most significant problems standing in the way of the broad commercial application of the stoichiometric LiMn_2_O_4_ spinel are a large polarization at high current rates and a considerable loss of capacity during subsequent charge and discharge cycles [10,11]. The poor cycling performance of LMO spinel can be ascribed to a reversible phase transition from cubic to orthorhombic near room temperature, derived from the Jahn–Teller distortion of Mn^3+^ ions [12,13,14], as well as partial manganese dissolution from the spinel according to the disproportionation of Mn^3+^ into Mn^2+^ and Mn^4+^ [15,16,17]. In order to surmount these obstacles, strategies including cation or anion doping [18,19,20], surface modification [21,22,23], and preparation of nanostructured spinels [24,25,26] were investigated. In our previous work, we revealed that sulphur substitution in the oxygen sublattice of lithium manganese oxide spinel can also be successfully implemented to promote its structural stability and electrochemical characteristic [27]. The LiMn_2_O_3.99_S_0.01_ (LMOS1) powder was nanocrystalline as prepared with a lattice parameter of about 0.826 nm. The value of the lattice constant for the LMOS1 sample—which is slightly higher than for LMO (a = 0.823 nm)—confirmed sulphur substitution for oxygen in the spinel structure, and is consistent with the previous studies published by Molenda et al. [28]. The substitution of sulphur into the LiMn_2_O_4_ spinel structure enabled suppression of the negative phase transition close to room temperature. Furthermore, the performed studies delivered an outstanding capacity and cycling behaviour of LiMn_2_O_3.99_S_0.01_ (LMOS1) cathode material in contrast to the stoichiometric LMO electrode. Nevertheless, to explain the enhanced cycling performance of the sulphided spinels, it is highly important to gain insight into the processes occurring within the cathode material and at the electrode/electrolyte interface. The leading method used to analyse the mentioned processes is electrochemical impedance spectroscopy (EIS), due to the differences in their time constants. 

Herein, to comprehend the improved electrochemical performance of sulphur-substituted spinel cathode material, detailed investigation of the electrochemical properties of LiMn_2_O_4-y_S_y_ materials (where y = 0 or 0.01 and they are called LMO and LMOS1, respectively) is presented. The impedance and diffusivity changes were determined on the basis of EIS measurements of Li/Li^+^/LMOS cells as a function of state of charge (SOC), settled at characteristic points during galvanostatic charging/discharging processes.

## 2. Materials and Methods

Nanostructured LMOS spinels (LMO and LMOS1 samples) were obtained using a modified sol–gel method based on the hydrolysis and condensation processes as previously described [27,29]. Briefly, for the preparation process, aqueous solutions of CH_3_COOLi·2H_2_O, (CH_3_COO)_2_Mn·4H_2_O, (NH_4_)_2_S, and NH_3_·H_2_O as the alkalizing agent were used. The syntheses were carried out with an argon flow to prevent oxidation of the Mn^2+^ ions. The formed sols were dried at 90 °C for 3–4 days, and then the obtained xerogels were calcined in air at 300 °C for 24 h and afterwards at 650 °C for 6 h. The structure and morphology of these materials have already been reported and can be found in our preceding work [27].

The electrochemical measurements of the synthesized spinels were carried out using R2032 coin-type cells. The Li/Li^+^/LMOS cells were assembled in an argon-filled glove box (MBraun Unilab, MBraun, Garching, Germany) with both H_2_O and O_2_ levels less than 0.1 ppm. The cathodes were fabricated by mixing 80 wt% of active material with 10 wt% of carbon black, used as conductive agent, and 10 wt% of polyvinylidene fluoride (PVDF) binder in *N*-methyl-2-pyrrolidone (NMP) solvent. The prepared slurry was stirred for 24 h and then coated on aluminum foil. Finally, after drying at 90 °C for the next 24 h, the cathode foil was pressed and cut into circular discs to form the working electrodes 12 mm in diameter. The typical loading of active materials in the assembled cells was around 1 mg·cm^−2^. As a negative electrode, a metallic lithium foil was used. Both electrodes were separated by a microporous polypropylene film (Celgard 2325, Celgard, Charlotte, NC, USA) and two porous glass microfiber filters (Whatman GF/F, Whatman, Maidstone, UK). The electrolyte was a 1 M solution of lithium hexafluorophosphate (LiPF_6_) in a mixture of ethylene carbonate (EC) and diethyl carbonate (DEC) at a volume ratio of 1:1. The galvanostatic charge and discharge tests (CELL TEST) were run at C/10 rate using ATLAS 0961 MBI multichannel battery tester (ATLAS-SOLLICH, Rębiechowo, Poland) at room temperature. Cut-off voltages were 4.5 and 3.0 V for the charge and discharge processes, respectively. Subsequent galvanostatic cycling, accompanied by electrochemical impedance spectroscopy, were conducted on a potentiostat/galvanostat AUTOLAB PGSTAT302N/FRA2 (Metrohm Autolab, Utrecht, The Netherlands). The EIS measurements were performed at different SOC of the cells by applying an alternating current signal of 0.005 V amplitude in the frequency range from 100 kHz to 0.01 Hz, every time after 30 min relaxation in a set potential. The impedance data was fitted using Nova 1.11 Autolab software based on the Boukamp model [30].

## 3. Results and Discussion

**Figure 1:**
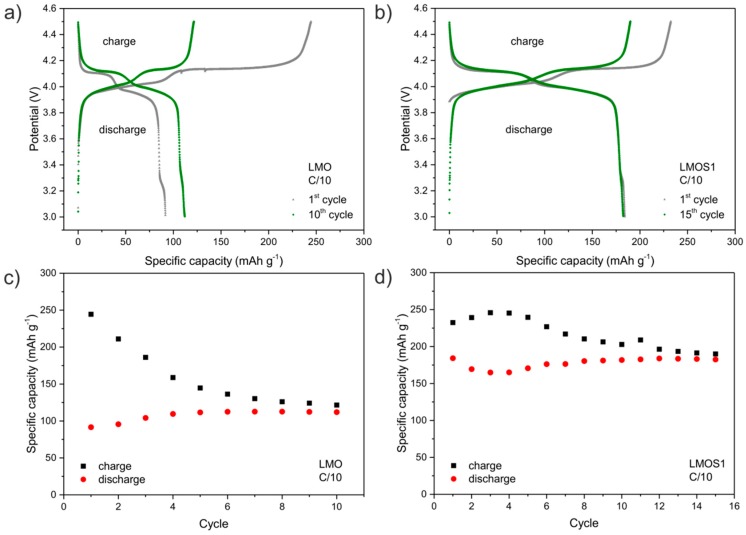
Galvanostatic charge-discharge voltage profiles for (**a**) lithium-manganese oxide (LMO) and (**b**) LiMn_2_O_3.99_S_0.01_ (LMOS1) cathode materials at C/10 current rate. Change in specific charge–discharge capacity as a function of cycle at C/10 rate of (**c**) LMO and (**d**) LMOS1 electrodes.

In order to investigate the electrochemical properties of LMOS spinel materials during a representative charge and discharge cycle, galvanostatic cycling between 3.0 and 4.5 V was performed until the stabilization of the initial electrochemical processes. In the first few cycles (10 cycles for LMO and 15 cycles for LMOS1), the additional charge is used in the passivation of the active materials. After the process is completed (after 10 or 15 cycles), the coulombic efficiency of the charge and discharge reaction remains constant for both materials. The results of the preliminary galvanostatic studies are presented in Figure 1.

The voltage profiles of the Li/Li^+^/LMO (Figure 1a) and Li/Li^+^/LMOS1 (Figure 1b) cells exhibit two distinct charge and discharge plateaus at around 4.0 and 4.15 V, which reflect a typical electrochemical behaviour of LiMn_2_O_4_ spinel [31]. It can be observed that the passivation process—which leads to the solid electrolyte interphase (SEI) layer formation—occurs simultaneously with the extraction/insertion of lithium ions in the LMOS systems in the whole potential range from 3.95 to 4.2 V. However, the passivation process of the LMOS1 material is more efficient, as reflected in the diminished irreversible capacity during initial cycles (Figure 1c,d). The specific discharge capacities of the studied materials—measured after completion of SEI generation—are 112 and 183 mAh g^−1^ for LMO and LMOS1, respectively. Although there are reports in the literature showing that the stoichiometric LiMn_2_O_4_ spinel can exhibit higher capacity than that demonstrated here (ca. 135 mAh g^−1^ [32,33]), these values are still reduced by about 35% with respect to the capacity of the resulting LMOS1 material, since it constitutes about 125% of the theoretical capacity for lithium manganese oxide spinel.

**Figure 2:**
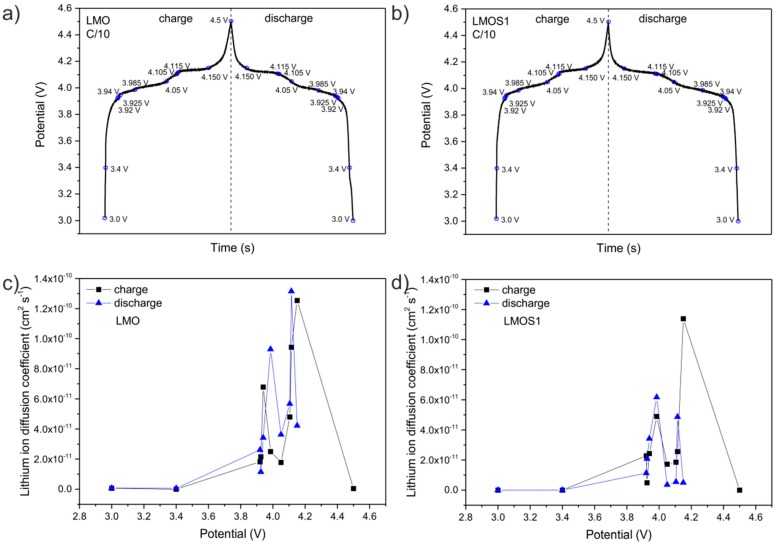
Voltage profiles of (**a**) LMO and (**b**) LMOS1 materials with marked potentials, in which electrochemical impedance spectroscopy (EIS) measurements were carried out. The lithium ion diffusion coefficients as a function of potential for (**c**) Li_x_Mn_2_O_4_ and (**d**) Li_x_Mn_2_O_3.99_S_0.01_, respectively.

After the preliminary galvanostatic cycling tests, the as-prepared samples were subjected to complementary analysis of changes in the electrochemical properties. This examination consisted of electrochemical impedance spectroscopy (EIS) measurements at different SOC during subsequent charge and discharge cycles. These characteristic potentials of EIS studies were selected based on the profile of the potential curves, which are presented in Figure 2a,b.

**Figure 3:**
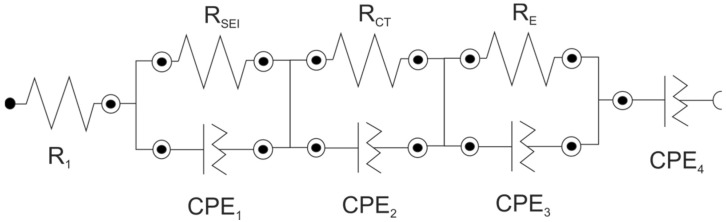
The equivalent circuit used to model the EIS spectra. CPE: constant phase element.

In accordance with the obtained results, the lithium ion diffusion coefficient (D_Li+_) was determined. The D_Li+_ was derived taking into consideration a finite-layer diffusion-impedance model (the so-called unsupported situation) [34,35]. The finite diffusion model is more realistic than the semi-infinite model, and is relevant for thin layers of solid materials. Generally speaking, in this case, the diffusion layer thickness (L_D_) is assumed to approach the sample thickness (d). For such a situation, the resulting finite length diffusion impedance response appears on a Nyquist plot at low frequencies as a diffusion tail, and is approximated by a constant phase element (CPE) (CPE_4_ in the equivalent circuit—Figure 3). Impedance of the CPE_4_ element can be further represented by a parallel combination of two separate elements—a diffusion capacitance (CPE_D_) and diffusion resistance (R_D_). The equation for diffusion resistance (according to which the Li^+^ diffusion coefficients were calculated) is expressed as [34]:

(1) $$R_{D}=\;\frac{RTL_{D}}{z^{2}F^{2}D_{Li+}AC}$$

where *R* is the gas constant; *T* is the room temperature; *z* is the number of the electrons per molecule taking part in the redox reaction; F is the Faraday constant; *A* is the surface area of the electrode, and *C* is the concentration of lithium ions. The relation of D_Li+_ versus potential for the LMOS samples is shown in Figure 2c (for LMO) and Figure 2d (for LMOS1). As demonstrated, the D_Li+_ values (disregarding the values calculated at the extreme points of potential) are in the range of 10^−12^ to 10^−10^ cm^2^ s^−1^ for both samples, which is in a good accordance with the previous studies published by Tang et al. [36]. Both of the compositional dependencies of D_Li+_ disclose two maximum peaks at 3.94, 4.15 V and 3.985, 4.115 V in the charge and discharge process, respectively, for LMO; and at 3.985, 4.15 V and 3.985, 4.115 V in the charge and discharge process, respectively, for LMOS1, which might exist as a result of higher diffusivity at the potentials corresponding to the two-step process of extraction/insertion of lithium ions in the spinel structure. The similar trend of diffusion coefficients with lithium content for lithium manganese oxide spinel was reported, for example, by Ouyang [37] and Saidi [38]. It has also been recognized that the lower diffusivity of lithium ions in the sulphided spinels does not contribute to the deterioration of electrochemical performance of LMOS1 spinel cathode material, as it shows significantly higher capacity and more stable cycling behaviour than the LMO electrode.

**Figure 4:**
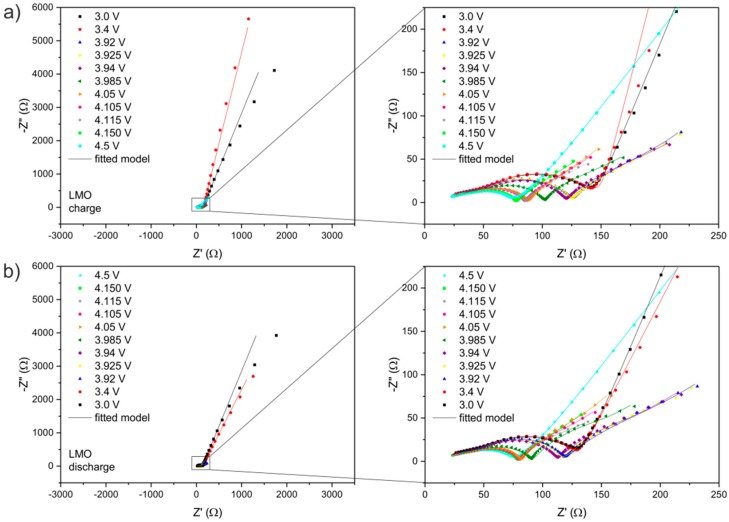
Nyquist plots for Li/Li^+^/LMO cell recorded at different potentials during (**a**) charge and (**b**) discharge. Z’ is the real part of impedance and -Z” is the imaginary part of impedance.

**Figure 5:**
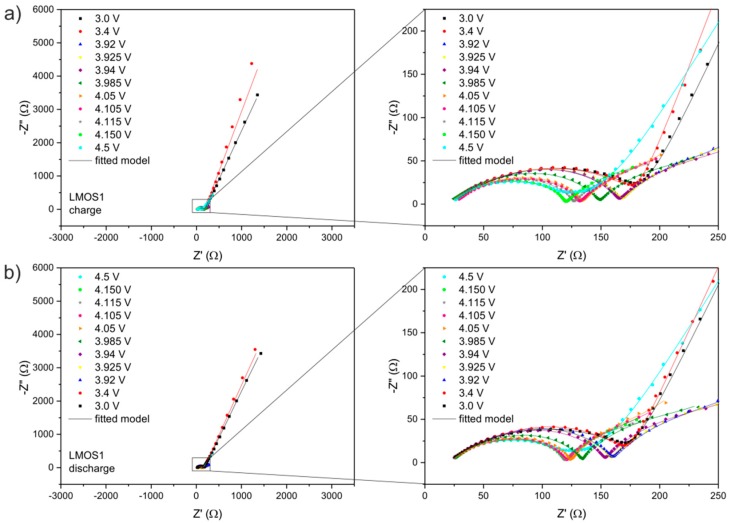
Nyquist plots for Li/Li^+^/LMOS1 cell recorded at different potentials during (**a**) charge and (**b**) discharge.

The set of Nyquist plots from EIS studies is illustrated in Figure 4 (for LMO) and Figure 5 (for LMOS1). The impedance spectra can be interpreted on the basis of the proposed equivalent circuit (Figure 3), the same for all curves. In this circuit, R_1_ refers to the uncompensated resistance of liquid electrolyte and the resistance between the electrode and the current collector. R_1_ corresponds to the shift of the plot along the real axis. The first parallel sub-circuit of R_SEI_/CPE_1_ elements is associated with the passivation layer properties, and its representation in the form of the depressed semicircle is included at the high frequency region of the distorted semicircle shown in the figures. The charge transfer reaction in the active materials is described by the second parallel sub-circuit (R_CT_/CPE_2_), which is responsible for the main part (at the high-to-medium frequency region) of the deformed semicircle. The last parallel connection of R_E_ and CPE_3_ in the equivalent circuit relates to the electronic properties of studied spinel cathodes [39,40]. The signal of these properties is visible on the spectra as a part of the depressed semicircle at the medium-to-low frequency region. The lithium ion diffusivity in the electrode materials (as already mentioned) is expressed in the circuit by the constant phase element (CPE_4_) connected in series and assigned to the tail at the low frequencies. The values of each resistor and constant phase element from the fitted circuit are given in Table 1 (for LMO) and Table 2 (for LMOS1).

**Figure 6:**
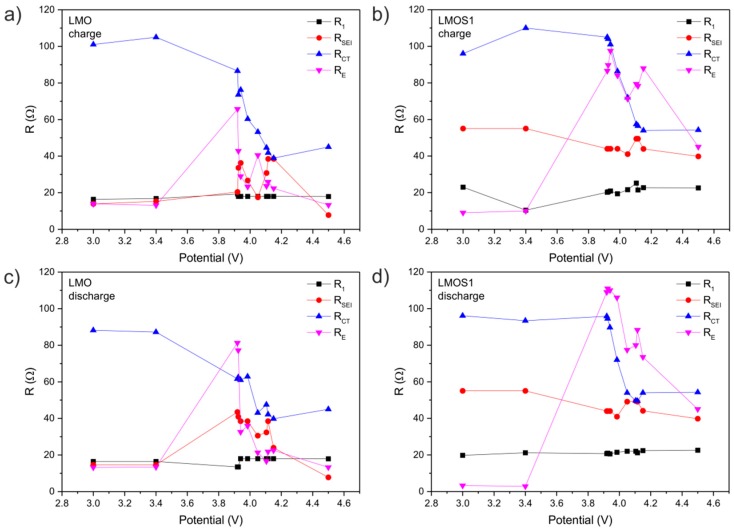
The changes of EIS parameters (calculated values of resistors in proposed equivalent circuit) for the stoichiometric spinel (LMO) during (**a**) charge and (**c**) discharge, as well as for the sulphided spinel (LMOS1) during (**b**) charge and (**d**) discharge.

The electrochemical behaviour of spinel materials at different states of charge were analysed based on the changes in the resistance of the particular elements of the equivalent circuit. The dependencies of resistances (R) versus cell potential are presented in Figure 6.

Firstly, it can be pointed out that the changes in ohmic resistance (R_1_) values are negligible (the resistances are ca. 20 Ω for all measurements). On the contrary, the resistance of SEI layers vary along charging/discharging process (i.e., lithium ion concentration and potential). As can be observed, the changes for the LMO sample are relatively greater than for LMOS1. The resistance of the SEI layer (R_SEI_) in LMOS1 is fairly stable during the electrochemical processes, and is in the range of 40–55 Ω. Despite the fact that the SEI resistance of LMO is lower at the beginning (15 Ω) as well as at the end (8 Ω) of the cycle, the large spikes (up to 44 Ω) in the plot of the R_SEI_ near 3.9 and 4.1 V are a direct indication of passivation layer instability. These effects occur during both charging and discharging, suggesting that the SEI layer may be constantly created and degraded upon the electrochemical reaction. In addition, its structure and composition may be changed, and this change usually results in the degradation of the performance of lithium cells. This behaviour comes from the reaction of active material with electrolyte, self degradation of active material structure, and decomposition of liquid electrolyte. The higher stability of the SEI layer formed on the surface of the sulphided spinel contributes to irreversible capacity reduction, improvement of the coulombic efficiency, and advancement of the cycle performance in comparison to the stoichiometric lithium manganese oxide spinel, confirming the results of our previous studies [27]. Even though the difference in the surface properties between stoichiometric and sulphur-substituted LiMn_2_O_4_ spinel has not been thoroughly determined, the obtained results clearly indicate that the enhanced performance of the LMOS1 material results from the greater stability of the SEI layer. The changes in the charge transfer resistance (R_CT_) are connected with the concentration of electron carriers in the structure. The charge transport in the lithium manganese spinel materials takes place through the small polaron mechanism, by the electron hopping between the two charge states of Mn^3+^ and Mn^4+^. While the lithium ions are deintercalated from the host material, the Mn^4+^/Mn^3+^ ratio increases, leading to a decrease in the amount of charge carriers. During discharging, this process is reversed. Slightly higher values of R_CT_ are observed for the LMOS1 sample. Hence, sulphur substitution can be considered to decline (to some extent) the diffusivity and electrical conductivity of the spinel material. The variation of electronic resistance (R_E_) along with the cell potential is cyclic and reversible. The R_E_ plots (concerning both charge and discharge processes) exhibit two separate peaks in the potentials of oxidation and reduction of active materials. This phenomenon can be explained by the inequality of manganese sites and the distribution of charge in the spinel structure. The greater changes in electronic resistance of the LMOS1 material may be related to the stronger distortion of the spinel network caused by the sulphur substitution [41].

## 4. Conclusions

The electrochemical properties of both stoichiometric and sulphur-doped lithium manganese spinel were successfully studied using electrochemical impedance spectroscopy carried out at different states of charge during representative charging/discharging cycles. Performed analysis revealed that the introduction of sulphur into the oxygen sublattice of spinel structure stabilizes the surface of the active material in relation to the reactivity with electrolyte compounds. The LMOS1 material is passivated during the first few charge and discharge cycles with a stable SEI layer, which ensures high coulombic efficiency and capacity retention, unlike the stoichiometric LMO. LMOS1 can provide stable reversible capacity of 183 mAh g^−1^ (under C/10 rate), which is ca. 125% of the theoretical capacity for LiMn_2_O_4_. Nonetheless, it should be pointed out that the increase of specific capacity and improvement in cyclability of sulphur-doped material appears at the expense of transport properties. In spite of that, we have every reason to assert that the addition of sulphur (≤0.01 mole of S) in the LiMn_2_O_4_ structure is advantageous. Most probably, a lattice distortion caused by the small amount of sulphur affects electronic structure of the material and generates a local density of states. The presented electrochemical impedance studies have not enabled complete understanding of the superior electrochemical performance of sulphided lithium manganese oxide spinels, and thus, further investigations including quantum mechanics calculations are required to adequately elucidate this issue. This research gives new insight into the electrochemistry of the modified spinel materials.

## Figures and Tables

**Table 1:** Parameters of EIS measurements (calculated values of resistors and constant phase elements in proposed equivalent circuit) for LMO electrode. R_1_, R_SEI_, R_CT_ and R_E_ stand for ohmic resistance, solid electrolyte interface (SEI) resistance, charge transfer resistance and electronic resistance respectively. Y_0_ and N are the parameters describing constant phase element (CPE).

	Li_x_Mn_2_O_4_	R_1_/Ω	R_SEI_/Ω	R_CT_/Ω	R_E_/Ω	CPE_1_		CPE_2_		CPE_3_		CPE_4_	
	Potential/V					Y_0_/Ω^−1^·s^N^	N	Y_0_/Ω^−1^·s^N^	N	Y_0_/Ω^−1^·s^N^	N	Y_0_/Ω^−1^·s^N^	N
**charging**	3	16	14	101	14	4.44E-06	0.840	4.00E-05	0.699	7.70E-07	0.846	2.24E-03	0.813
	3.4	17	15	105	13	8.60E-07	0.840	4.14E-05	0.699	5.21E-06	0.846	2.11E-03	0.885
	3.92	19	20	87	66	1.22E-06	0.841	4.22E-05	0.684	4.57E-02	0.696	7.99E-02	0.649
	3.925	18	34	74	43	1.30E-05	0.641	3.20E-05	0.756	5.24E-02	0.716	5.47E-02	0.576
	3.94	18	36	76	29	6.94E-02	0.834	6.00E-05	0.663	2.56E-05	0.591	6.15E-02	0.573
	3.985	18	27	60	23	1.19E-01	0.826	7.84E-05	0.515	2.50E-05	0.939	6.73E-02	0.522
	4.05	18	18	53	41	2.41E-06	0.796	5.36E-05	0.664	9.00E-02	0.726	1.13E-01	0.677
	4.105	18	31	45	24	1.15E-01	0.755	5.94E-05	0.685	1.23E-05	0.668	1.04E-01	0.619
	4.115	18	39	42	26	9.10E-02	0.708	4.65E-05	0.728	1.20E-05	0.668	1.33E-01	0.619
	4.15	18	39	39	22	1.21E-01	0.675	5.14E-05	0.707	1.21E-05	0.668	1.27E-01	0.619
	4.5	18	8	45	13	6.85E-03	0.840	3.95E-05	0.699	1.60E-06	0.846	1.75E-02	0.653
**discharging**													
	4.15	18	24	40	23	1.44E-01	0.792	7.50E-05	0.668	2.42E-05	0.631	9.58E-02	0.619
	4.115	18	39	42	22	9.57E-02	0.797	1.04E-04	0.515	1.66E-04	0.668	1.32E-01	0.619
	4.105	18	32	48	17	1.01E-01	0.817	7.83E-05	0.632	1.47E-05	0.668	9.34E-02	0.619
	4.05	18	31	43	21	9.80E-02	0.809	5.64E-05	0.693	1.56E-05	0.653	6.72E-02	0.622
	3.985	18	39	63	36	8.36E-05	0.675	4.55E-02	0.751	1.10E-04	0.654	1.01E-01	0.644
	3.94	18	39	61	33	5.35E-02	0.760	2.62E-05	0.780	1.09E-05	0.659	4.21E-02	0.515
	3.925	14	41	63	77	2.94E-05	0.557	2.74E-05	0.780	3.35E-02	0.652	6.25E-02	0.598
	3.92	14	44	62	81	3.70E-05	0.536	2.66E-05	0.788	3.23E-02	0.635	6.02E-02	0.600
	3.4	17	15	87	13	8.49E-07	0.840	3.96E-05	0.699	4.83E-06	0.846	3.03E-03	0.772
	3	17	15	88	13	8.49E-07	0.840	3.96E-05	0.699	4.83E-06	0.846	2.29E-03	0.808

**Table 2:** Parameters of EIS measurements (calculated values of resistors and constant phase elements in proposed equivalent circuit) for LMOS1 electrode.

	Li_x_Mn_2_O_3.99_S_0.01_	R_1_/Ω	R_SEI_/Ω	R_CT_/Ω	R_E_/Ω	CPE_1_		CPE_2_		CPE_3_		CPE_4_	
	Potential/V					Y_0_/Ω^−1^·s^N^	N	Y_0_/Ω^−1^·s^N^	N	Y_0_/Ω^−1^·s^N^	N	Y_0_/Ω^−1^·s^N^	N
charging	3	23	55	96	9	9.03E-05	0.805	2.40E-05	0.691	7.57E-06	0.763	2.50E-03	0.784
	3.4	10	55	110	10	8.40E-05	0.568	5.98E-05	0.664	6.74E-09	0.959	2.26E-03	0.827
	3.92	20	44	105	87	6.83E-05	0.575	3.30E-05	0.722	3.30E-02	0.728	9.77E-02	0.681
	3.925	21	44	104	90	4.87E-05	0.603	3.04E-05	0.737	3.02E-02	0.721	1.05E-01	0.691
	3.94	21	44	101	98	4.73E-05	0.605	2.92E-05	0.742	3.17E-02	0.701	1.23E-01	0.664
	3.985	19	44	86	84	5.77E-05	0.590	3.18E-05	0.734	4.15E-02	0.705	1.88E-01	0.686
	4.05	22	41	72	71	4.27E-05	0.621	3.36E-05	0.737	5.59E-02	0.707	1.81E-01	0.729
	4.105	25	50	57	79	36.2E-06	0.631	3.47E-05	0.767	5.85E-02	0.675	2.00E-01	0.689
	4.115	22	50	57	78	3.96E-05	0.624	3.55E-05	0.763	5.81E-02	0.685	2.56E-01	0.698
	4.15	23	44	54	88	3.42E-05	0.643	3.84E-05	0.751	6.75E-02	0.662	3.13E-01	0.649
	4.5	23	40	54	45	9.71E-05	0.694	3.37E-05	0.650	6.51E-03	0.501	1.57E-02	0.733
discharging													
	4.15	22	44	54	74	3.81E-05	0.635	3.81E-05	0.751	6.21E-02	0.690	2.40E-01	0.735
	4.115	21	49	50	88	3.74E-05	0.631	3.30E-05	0.786	5.28E-02	0.698	2.91E-01	0.715
	4.105	22	50	50	80	3.70E-05	0.632	3.22E-05	0.790	5.08E-02	0.703	1.95E-01	0.733
	4.05	22	49	54	77	4.27E-05	0.619	3.19E-05	0.780	4.73E-02	0.744	1.47E-01	0.752
	3.985	22	41	72	106	4.23E-05	0.623	2.85E-05	0.758	3.44E-02	0.725	1.76E-01	0.710
	3.94	21	44	90	110	5.54E-05	0.594	2.77E-05	0.747	2.80E-02	0.723	1.24E-01	0.687
	3.925	21	44	95	111	4.65E-05	0.608	2.66E-05	0.751	2.75E-02	0.706	1.02E-01	0.671
	3.92	21	44	96	109	4.71E-05	0.607	2.67E-05	0.750	2.71E-02	0.703	9.42E-02	0.677
	3.4	21	55	93	3	2.40E-05	0.678	3.81E-05	0.747	8.03E-07	0.983	2.48E-03	0.792
	3	20	55	96	3	2.10E-05	0.678	4.45E-05	0.747	2.71E-07	0.983	2.48E-03	0.792
